# Sample Size Calculation in Genetic Association Studies: A Practical Approach

**DOI:** 10.3390/life13010235

**Published:** 2023-01-14

**Authors:** Cristina Politi, Stefanos Roumeliotis, Giovanni Tripepi, Belinda Spoto

**Affiliations:** 1 Clinical Epidemiology and Renal Diseases and Hypertension Unit, Institute of Clinical Physiology (IFC), National Research Council (CNR), 89124 Reggio Calabria, Italy; 2 Division of Nephrology and Hypertension, 1st Department of Internal Medicine, AHEPA Hospital, School of Medicine, Aristotle University of Thessaloniki, 54636 Thessaloniki, Greece

**Keywords:** genetic association study, SNP, sample size, genetic software, complex diseases, diabetic nephropathy

## Abstract

Genetic association studies, testing the relationship between genetic variants and disease status, are useful tools for identifying genes that grant susceptibility to complex disorders. In such studies, an inadequate sample size may provide unreliable results: a small sample is unable to accurately describe the population, whereas a large sample makes the study expensive and complex to run. However, in genetic association studies, the sample size calculation is often overlooked or inadequately assessed for the small number of parameters included. In light of this, herein we list and discuss the role of the statistical and genetic parameters to be considered in the sample size calculation, show examples reporting incorrect estimation and, by using a genetic software program, we provide a practical approach for the assessment of the adequate sample size in a hypothetical study aimed at analyzing a gene–disease association.

## 1. Introduction

The prevalence of hypertension, diabetes, heart failure and cancer is continuously rising worldwide [1]. These complex clinical conditions are influenced by the function of multiple genes that, along with environmental factors, induce the development and/or progression of these morbid conditions. In this setting, a risk variant is not deterministic like in Mendelian monogenic disorders but only increases the risk of disease, typically contributing a weak effect on the etiology of the disease itself [2].

Genetic association studies, analyzing the relationship between genetic variants and disease traits, are a useful tool for detecting susceptibility variants that contribute to complex diseases [3]. In this respect, if a genetic variant occurs more often than expected by chance in affected subjects, then it could be suggested that this variant is associated with an increased risk for the disease [4]. During the last two decades, the Human Genome Project, by providing a deeper comprehension of human diversity, provided a great boost to such studies, allowing us to exploit genetic variants, i.e., microsatellites, insertions/deletions, variable number tandem repeats (VNTRs), copy-number variants (CNVs) and single nucleotide polymorphisms (SNPs), as essential tools to unravel the genetics of multifactorial disorders [5]. Even just focusing on SNPs, the most common type of genetic variant, the likelihood of identifying numerous genetic determinants that contribute to the pathogenesis of rare and common diseases is high [6]. Indeed, although the human genome variable portion is less than 0.1%, it is equivalent to several million nucleotide differences per individual. However, to date, despite the fact that most genetic association studies are adequately designed and analyzed [7], there are some studies in this field that have not completely fulfilled their promises for the inconsistency of scientific data, mainly due to inadequacies in study designs [8,9,10]. In particular, an inadequate number of patients is one of the major and most common limitations in these studies, thus demonstrating that sample size is a crucial factor for the success of a study [2,11,12]. Indeed, a small sample may include a disproportionate number of outlier subjects that skew the fair picture of the population, whereas too large a sample makes the study complex, expensive and time-consuming to run. Thus, selecting the correct sample size is the only way to ensure reliable results.

With this perspective, we will focus on the thorny issue of sample size in genetic association studies in unrelated individuals and analyze the parameters to be considered for determining the adequate number of subjects to enroll. Then, we will examine tools designed for sample size calculation in genetic association studies and apply them to examples from the nephrology literature to critically evaluate the reliability of the results.

Despite the relevant role of gene–gene and gene–environment interactions in the comprehension of the gene–disease link, we will overlook the issue because it is beyond the aim of the manuscript.

## 2. Sample Size Definition and Parameters for Its Calculation

When the number of subjects in a population is too large to be studied, the investigation is often restricted to a subset of manageable size of randomly collected individuals. As a consequence of random sampling, virtually no difference exists regarding the characteristics between individuals from the sample and those from the source population; thus, any conclusion in the sample can be inferred to apply to the entire population. However, the fact that we test hypotheses in a sample rather than in the population poses a problem of agreement between the results observed in the sample and those observed in the population. In the source population, a given effect may not exist or exist, thus raising two mutually exclusive hypotheses, the null hypothesis (H_0_) and the alternative hypothesis (H_1_). To put it simply, in genetic association studies, H_0_ asserts that a variant is not associated with the phenotype of interest, whereas H_1_ states the opposite.

By definition, the sample size is the number of subjects included in a study to represent the real population. The greater this number, the more representative it is of the population as a whole. However, “*bigger is not better*” and the assessment of the adequate number of individuals needed to answer the research question is crucial to avoid wasting time and resources [13]. The process of sample size calculation is known as power analysis and has the purpose of determining the smallest sample size suitable to detect the occurrence of a “true” effect that is necessary to reject the null hypothesis. Normally, power analysis should be carried out before the data collection, at the design stage of a quantitative study, but it can also be formally performed during the study (adaptive design—sample size reassessment) if no reliable information on parameters is available in the planning phase [14].

The sample size calculation depends on several parameters whose values, if inappropriately assumed, determine the inconsistency of the results. Although the probabilistic nature is the intrinsic limitation of this procedure, the power analysis is the unique strategy that allows researchers to grant robustness to the studies.

In the genetic field, the associations between genes and complex diseases can reliably be detected and replicated if the sample size is appropriately large. In this setting, the sample size calculation is a complex question that includes a number of assumptions about a series of basic and specific parameters such as the alpha and beta errors, the power of the study, the variability of the outcome, the prevalence of the disease, the effect size, the allele frequency, the strength of linkage disequilibrium, the genetic models and the misclassification errors (Table 1).

### 2.1. Alpha and Beta Errors

In the planning phase of a genetic association study, two errors, namely the Type I error (or alpha) and the Type II error (or beta), should be considered.

These errors measure the probability of incorrectly rejected or retained H_0_, generating false positive or false negative results, respectively.

Specifically:

- Alpha (α) or the probability of Type I error occurs when H_0_, although actually true in the source population, is falsely rejected based on sample results (false positive result) [14]. The magnitude of this error is chosen in advance and conventionally fixed at 0.05 or 5%.
- Beta (β) or the probability of Type II error occurs when H_0_, although false in the source population, is incorrectly not rejected according to sample results (false negative result) [14]. It is usually set at 0.20 or 20%.

Power is the complement of beta, i.e., 1 − β or Type II, error probability. Consequently, if β is 0.20, the power is 0.80, meaning that there is 80% probability of avoiding a false negative result or the chance of correctly detecting a significant difference in the sample when it actually exists in the source population [15].

As a general rule, the greater the number of subjects, the lower the errors (α and β), the higher the study power and the more robust the study conclusions.

### 2.2. Outcome

The outcome is the dependent variable of a study that changes in relation to one or more independent variables. Based on the number of values that it can take, this variable may be categorical (limited values) or continuous (infinite values). In genetic association studies, if the outcome is a binary variable (i.e., presence or absence of the disease), the parameter to be considered for the calculation of the sample size is the prevalence of the disease, which is the proportion of individuals with the disease at a given point in time. If the outcome is a continuous variable (i.e., systolic blood pressure, body mass index), the parameter used to assess the sample size is the estimate of its variability, i.e., the standard deviation (SD). The greater the SD, the larger the sample size needed to obtain that an observed effect is actually true. When the prevalence of the disease and the variability of the outcome in the population of interest are unknown, these data may be retrieved from the literature.

### 2.3. Effect Size

The effect size is a numeric value that measures the magnitude (or size) of the relationship between two variables or the difference between groups as it would be expected to occur in a population [16]. The larger the effect size, the stronger the relationship between two variables or the greater the difference between groups. The effect size is essential to interpret results that, despite being statistically significant, may have such a small effect that they are considered clinically irrelevant. Specifically, in genetic association studies, the effect size measures the strength of the association between a gene variant and a disease or the magnitude of the difference in the allelic/genotypic frequency between groups (i.e., cases and controls).

For binary outcomes, it is expressed as a relative risk to odds ratio depending on the study design, while for continuous outcomes, as a percentage of the phenotypic variance. An odds ratio of three indicates triple odds of disease, and an effect size of 25% means that a quarter of the variability of a continuous trait is attributable to the risk allele.

The effect size and the sample size are two measures inversely related; the larger the effect size, the smaller the number of patients to be studied [16]. Nevertheless, the genetic effect on complex diseases is usually modest and characterized by relative risks/odds ratios of 1.1–1.5 (a 10–50% increase in the probability of developing a disease) and a contribution to a continuous genetic trait of about 0.05% [17]. Such small effects explain, at least in part, why large samples are usually needed to perform reliable genetic association studies.

### 2.4. Minor Allele Frequency

A single nucleotide polymorphism is a genetic variant present in at least 1% of the population. It results from a single nucleotide substitution at one specific locus in the genome and provides two alternative forms of a gene, known as alleles [18]. Depending on the DNA regions where they fall, SNPs may change the amino acid sequence of proteins or affect the expression levels of the genes. However, independently of their functional role, SNPs are crucial for the inter-individual variability of the human genome and, as a consequence, the person’s genetic makeup that is predisposed to diseases [19]. According to the frequency of the minor allele (MAF), a genetic variant can be classified as common (MAF > 5%), uncommon (1–5% MAF) and rare (MAF < 1%). Typically, genetic association studies are sufficiently powered only to test common genetic variants because uncommon and rare variants are found in a small number of individuals and need of huge samples to be studied. For the most complex diseases, the allele with the least frequency implies risk more often than protection in populations of European ancestry [20]. However, since allele frequencies vary considerably across populations [21], a variant which is common in a population may be rare in another one, making the sample size assessment a population-dependent calculation.

### 2.5. Linkage Disequilibrium

Linkage disequilibrium (LD) is the non-random association of two or more alleles at different loci that occur together on the same haplotype more often than expected. Alleles in high LD are closely linked on a chromosome and thus are seldom separated by recombination events. They can be grouped into clusters of variable size, termed LD blocks, which are inherited as such and whose pattern varies across the populations depending on the genetic processes which concurred to structure the genome of each population. The standard pairwise measures of LD are D’ and r^2^, namely Lewontin’s coefficient and the equivalent to the Pearson correlation coefficient, respectively [22]. Both these coefficients measure the strength of the association between alleles and range from 0 to 1, that is, from unlinked alleles to alleles in complete LD. In common practice, r^2^ is more widely utilized, and the threshold of r^2^ > 0.8 is used to assume that two alleles are strongly associated.

The power of a genetic association study is a function of LD, being maximal if the causal variant and the marker SNP are in complete LD. Otherwise, if the causal variant and the marker SNP are incompletely associated, the power decreases parallel to the reduction of LD between the disease and the marker allele. Therefore, the stronger the correlation of the marker allele to disease allele, the higher the power of the study and the lower the sample size required to detect the gene–disease link.

Some genetic association studies consider a set of variants, called tag SNPs, that are the minimum number of SNPs able to capture the whole variability of the gene in strong linkage disequilibrium with all the other SNPs mapping on the gene itself. These studies require testing multiple associations; then, to avoid increasing alpha error and false positive results, a correction for multiple comparisons and, consequently, a proportional enhancement in the size of the sample are recommended steps.

### 2.6. Genetic Inheritance Model

The model of inheritance of a genetic variant is often unknown; then, for the assessment of the clinical effect of an SNP, a genetic model needs to be assumed [23]. Three genetic models are usually adopted: a dominant, recessive and additive model. In the dominant model, the *A* allele is assumed to prevail on the *a* allele, thus generating two genotypes, *AA* and *Aa*, that, identically influencing the risk of disease, are grouped in a single category, opposed to the one including the aa genotype (*AA* + *Aa* vs. *aa*). Conversely, in the recessive model, the aa genotype is the only one with clinical relevance, compared to *Aa* and *AA* genotypes, which are then both included in a single group, providing again a binary model (*aa* vs. *Aa* + *AA*). Finally, in the additive model, the dichotomization of the genotypes is lost in favor of an independent categorization of the three genotypes that are assumed to contribute differently to phenotype (*AA* vs. *Aa* vs. *aa*).

The additive model is frequently assumed when there is no biological evidence regarding the underlying genetic model of an SNP [24]. However, misspecifying the true genetic model results in a reduction of the study power [25]. Indeed, if a binary (dominant or recessive) model is incorrectly modelled as additive, the risk of disease for heterozygous individuals is under- or overestimated, and this imprecision represents an error that decreases the power of the association. Of note, the magnitude of the error changes in relation to the frequency of the SNP because the more common the SNP, the larger the proportion of heterozygotes and, hence, the number of individuals with an inaccurate estimate of the risk. Therefore, at the design stage of a genetic association study, the most conservative sample size under the three genetic models should be selected in order to ensure adequate power, even when a misspecification error can occur.

As a general rule, under the same assumptions, the dominant model needs the smallest sample size to achieve adequate statistical power, whereas the recessive model is the one that requires the largest number of participants.

### 2.7. Errors

Genotype/phenotype misclassification and missing genotype data are common errors in genetic association studies and their effect on the power loss is, actually, impossible to quantify at the design stage of the study. Although statistical strategies may be adopted to deal with such errors, a convenient solution is to design a study with a higher power [26], though the improvement of data quality remains the best approach to minimize errors and limit the number of individuals enrolled in the study [27].

## 3. Examples of the Sample Size Calculation

    The calculation of the sample size should be performed for all genetic association studies and each assumption underlying the power analysis should be accurately reported. However, the majority of studies indicate only the power and the alpha error values, completely neglecting to show the estimates of all the parameters conventionally used for sample size calculation. In particular, in nephrological research, studies designed to test the effect of gene variants on renal diseases rarely include the sample size calculation. To assess the prevalence of power analysis in genetic studies in nephrology populations, we carried out a search of Medline, as shown in Box 1. In the overwhelming majority of the retrieved articles (N = 1660), sample size calculation was not reported. Specifically, the proportion of the studies including the power analysis was 15/1660 (0.9%) (Box 1); this result provides evidence that, although the number of studies investigating the effect of SNPs in renal disease is relevant, the sample size calculation is an extremely overlooked issue.

Box 1 **Search strategy and selection criteria.**

In a search strategy aimed at identifying the genetic association studies in patients with kidney disease that include power analysis, we searched Medline database for the effect of SNPs on various renal outcomes in case-control and prospective studies. We identified papers combining the following search terms in the title: “genotype” or “polymorphism” or “isoform” or “genome-wide” or “GWAS” or “genetic” or “allele” or “gene” AND “kidney” or “renal” or “dialysis” or “hemodialysis” or “nephropathy” or “transplant” or “glomerular filtration rate” or “allograft” AND “death” or “mortality” or “cardiovascular” or “myocardial infraction” or “stroke” or “peripheral arterial disease”. After removing duplicates, the research produced 1660 articles and only 15 studies included the power analysis in the Methods section.

Of the 15 retrieved studies, 5 included a post hoc power analysis, 4 only reported a brief sentence about the sample size calculation without specifying the parameters considered to perform this analysis and 3 were GWAS studies reporting poor information about the power analysis. The remaining 3 were the studies that we took into account for our didactic purposes.

### 3.1. Example 1

The study by Yang et al. analyzed the association between the Pro12Ala polymorphism in the peroxisome proliferation-activated receptor γ 2 (PPARγ2) gene and the progression of diabetic nephropathy in patients with type 1 diabetes [28]. The authors genotyped 197 Caucasian patients with type 1 diabetes (116 with diabetic nephropathy and 81 without renal complications) and 151 ethnically matched healthy controls and found no association between the Pro12Ala polymorphism and the rate of decline in renal function in terms of estimated glomerular filtration rate (GFR).

The power of the study was assessed by a post hoc analysis carried out by UCLA Binomial power calculation software. The analysis shows that the sample size reported in the study had a 75% power, at a 0.05 significance level, to detect a difference of 2 mL/min/year in the rate of GFR decline between the two genotype groups (Pro12Pro vs. Pro12Ala + Ala12Ala) of the Pro12Ala polymorphism in diabetics and healthy controls, as well as in diabetic patients with and without nephropathy.

#### Criticisms

In this study, the power calculation has several flaws. First and foremost, the UCLA Binomial power calculation software is inadequate to assess the study power in genetic association studies because it lacks any information about a series of genetic parameters that strongly influence this calculation. Second, the authors do not provide any details about the expected effect size in the two groups, actually making the power calculation unreproducible. Third, a study with a power less than the conventional threshold of 80% is usually considered quite unreliable [29].

### 3.2. Example 2

Scientific evidence supports a key role for some allelic variants located in genes involved in the immune response and bioavailability and kinetics of immunosuppressive therapy in acute rejection [30]. Screening for these SNPs prior to transplantation may provide valuable information to clinicians who can tailor personalized drug dosage to ensure maximum immunosuppression with minimum toxic side effects.

On this background, Scalzotto et al. tested the association between 19 SNPs within five different genes (IL10, TNFα, ABCB1, UGT1A9 and IMPDH2) involved in the inter-individual variability in immunosuppressive treatment response and the incidence of acute rejection in kidney transplant (KT) patients [31]. They enrolled 220 subjects and divided them in three experimental groups: 41 KT recipients with acute rejection (Case group), 109 KT recipients without acute rejection (Control I group) and 70 healthy blood donors (Control II group). Genetic analyses revealed that patients with C allele at rs1045642 and A allele at rs2032582 of the ABCB1 gene were at high risk for acute rejection after transplantation.

#### Criticisms

In this study, the issue of the sample size is shortly addressed in the Statistical Analysis paragraph where the authors report having performed power analysis using the QUANTO program. With the exception of α = 5% and power (1 − β) = 80%, the authors do not include any further information about the parameters used to estimate the sample size, making this process unreproducible.

### 3.3. Example 3

This study is an extension of the study reported above and was carried out by the same authors after having increased the number of KT recipients with acute rejection (Case group) from 41 to 74 patients [32]. The size of the Control I and Control II groups remained unchanged, and the total number of enrolled individuals was 253. The aim of this new study was to reassess the genetic associations previously observed in light of a larger Case group, which was underpowered in the preliminary study. The results of the current analysis did not confirm the associations between the polymorphic variants in the ABCB1 gene and the risk of acute rejection after transplantation.

#### Criticisms

In this study, the crucial role of the sample size is clearly addressed, mentioned in the title and extensively discussed in the paper. The authors claim that the sample size calculation is a fundamental but usually neglected aspect of the scientific research design, highlighting it as the reliability of study results may be compromised by an inadequate sample size. However, they do not report any information about the procedure applied to assess the sample size. In particular, they do not provide any important parameter, such as the prevalence of acute rejection in KT patients or the effect size of the SNPs on acute rejection in renal allograft recipients, limiting themselves to stating in the Methods section that, according to the QUANTO program, the number of cases and controls was adequate to achieve an 80% study power at the significance level of 0.05.

## 4. Sample Size Calculation: A Practical Approach

Neuropeptide Y (NPY) is a 36 amino-acid peptide with various physiological actions present in both central and peripheral nervous systems. It is a strong vasoconstrictor and is abundantly released from post-ganglionic sympathetic neurons in response to sympathetic stimulation [33]. Chronic kidney disease is a condition characterized by high sympathetic activity, whose detrimental effects on blood pressure and glomerular filtration rate (GFR) are partly mediated by NPY [34]. NPY derives from the pre-proneuropeptide NPY (prepro-NPY) that, by undergoing a series of enzymatic cleavages, yields the mature molecule [35]. The genetic polymorphism 1128T>C (rs16139) in the signal sequence of the prepro-NPY causes an amino acid change from leucine to proline at codon 7 (Leu7Pro) that, by altering intracellular processing of the precursor, increases NPY bioavailability [36]. The functional role of this polymorphism is consistent with higher plasma levels of NPY in Pro7 carriers in response to maximal stress conditions [37].

In European countries, the frequency of the C allele (Pro7) increases according to a geographical south-to-north gradient [38], and in Swedish female patients with type 1 diabetes, this allele has been significantly associated with diabetic nephropathy [39].

As an example, we will perform a power analysis aimed at determining the smallest sample size suitable to detect whether such an association holds true in CEU patients with type 1 diabetes as well. For this purpose, we will use the QUANTO program [40] and illustrate, step-by-step, the parameter settings required by the software to test the main effect of the NPY 1128T>C polymorphism on diabetic nephropathy in a case-control study.

We open the calculator, choose Parameters in the toolbar on the top of the screen, and proceed as follows:

### 4.1. Outcome/Design (Figure 1)

The study is aimed at comparing the genotype frequencies of the 1128T>C polymorphism between diabetic patients with renal complication (cases) and those without it (controls). Therefore, we select Disease and not Continuous outcome, because our outcome is a binary variable (having/not having the disease), and choose an unmatched case-control design. In the Controls per Case dialog, we type the value of 1, meaning that we choose an equal number of controls and cases. In this respect, by increasing the number of controls per case, the study power linearly increases up to a control-to-case ratio of 4:1 [41].

### 4.2. Hypothesis (Figure 2)

We select Gene Only because we are interested in the main effect of the gene on the disease and not in the gene–environment or gene–gene interaction.

### 4.3. Gene G (Figure 3)

In the dialog, under Allele Frequency, we type the frequency of the C allele; that is 0.04 (4%) in CEU population. Sometimes the frequency of the minor allele may vary across subpopulations belonging to the same ethnic group and, thus, the boundary values should be typed together with a fixed incremental rate.

In the same dialog, under Inheritance mode, we select the dominant model for the C allele, given that in CEU population the CC (Pro7/Pro7) genotype is extremely rare.

The values reported in the Susceptibility Frequency box are automatically computed based on the allele frequency and inheritance model selected.

### 4.4. Outcome Model (Figure 4)

In the Disease Risk Parameters dialog, we select Population Risk and type 0.30 (30%); that is the prevalence of the disease (Kp) in the population and, specifically, the risk of diabetic nephropathy in CEU patients with type 1 diabetes, as retrieved from the literature [42].

Then, in the Genetic Effect box, we type the genetic relative risk/odds ratio (Rg); that is 2.614, as reported by Ma et al. [39]. As for the minor allele frequency, the size of the genetic effect may also have a precise value or range between two values.

The OR Summary box automatically reports the genetic effect size according to the genetic model assumed, where 1.0 is the reference risk and 2.614 the risk of disease that is associated to the C allele.

### 4.5. Power (Figure 5)

In the Power dialog, the arbitrary value of 0.80 is automatically reported, as well as the value of 0.05 for the significance level (Type I error rate) of a two-tailed statistical test. These values can be freely modified according to stringency conditions.

### 4.6. Calculate (Figure 6)

The Calculate selection provides a table reporting the number of case-control pairs (N) needed to achieve the desired power.

If the genetic effect size ranges between two values, the output provides a table including a list of sample size values that decrease parallel to the increase of the effect size.

**Figure 7:**
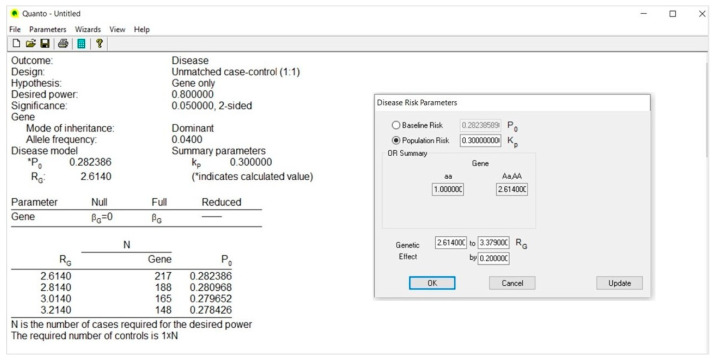
Calculate (arbitrary incremental rate).

Specifically, if we consider that the effect size may vary between 2.614 and 3.379 [39] and type this interval in the Genetic Effect box using an arbitrary incremental rate of 0.20, we obtain a table including four values of sample size, each calculated according to a corresponding value of effect size (Figure 7).

## 5. Software Programs for the Sample Size Calculation

Various software programs for sample size calculation in genetic association studies are currently available. They can be used to calculate the sample size based on different types of data and study designs. Several programs require a paid license but there are also some websites that allow sample size calculation for free. Examples of freely available programs are:

- QUANTO. It is a program that allows one to address hypotheses related to gene and environment only; gene–environment and gene–gene interactions. It is applicable to discrete and continuous phenotypes and deals with a large range of study designs including matched and unmatched case-control studies, case-sibling, case-parent trio and case only. In addition to the basic parameters, it requires specific parameters such as the MAF, the disease prevalence, the genetic effect size and the inheritance model [40].
- ESPRESSO.G. It is an R script sample size calculator aimed at investigating the role of genes and environment on discrete and continuous phenotypes. It supports calculations for stand-alone case-control studies and for case-control analyses nested in cohort studies. In particular, this tool takes into account genotype/phenotype misclassification errors [43].
- GENPWR. It is an open-source R package that defines the sample size for dichotomous or continuous outcomes in gene only and gene–environment interaction studies. By comparing the various genetic models (additive, dominant and recessive) to find the one that best fits the data, this tool allows one to calculate sample size in the presence of genetic model misspecification [44].
- OSSE (Online Sample Size Estimator). It is the simplest online sample size estimator for case-control study design. The unique parameters required are the alpha error, the study power, the MAF for cases and controls and the ratio of cases to controls. No assumptions about the nature of the phenotype, the genetic inheritance model or the effect size are needed. This software does not address gene–environment interactions [16].
- PAWE (Power for Association With Error). It is a sample size calculation web-based tool available on the Rockfeller site and designed for genetic case-control studies that evaluate the association between SNPs and complex discrete traits. Among the specific parameters, it requires the effect size, the MAF and the LD and allows for misclassification errors [45].
- GPC (Genetic Power Calculator). It is a user-friendly computer software that performs power calculations for genetic association studies. It computes statistical power under varying disease allele frequency, disease prevalence, genotype relative risk and inheritance models. This tool requires a priori the number of cases and controls and graphically plots the relation between sample size and study power [46].
- PGA (Power for Genetic Association analyses). It is a package tailored to estimate sample size in case-control studies of SNPs in unrelated individuals. However, being able to account for statistical multiple comparisons, it can also be used for fine-mapping association studies and whole-genome scans. The program provides a computational and graphical interface for the relation between statistical power and sample size under different genetic inheritance models, disease allele frequency, disease prevalence and relative risk and accounts for LD between the marker and the disease allele [47].

## 6. Conclusions

The sample size critically affects the reliability of scientific studies. If the sample is too small, the results may not accurately describe the population, while if it is too large, the study may become unwieldy and wasteful in terms of time and resources. Therefore, planning an accurate estimation of the sample size at the design stage of a genetic association study is crucial to obtain consistent results. Unfortunately, in the nephrology field of the genetic research, the sample size calculation is an overlooked issue being rarely performed and, when provided, it is often inadequately reported. In this context, the knowledge of the parameters that play a role in sample size calculation and the software programs developed for its estimation are essential components for improving the quality of scientific research.

## Figures and Tables

**Figure 1:**
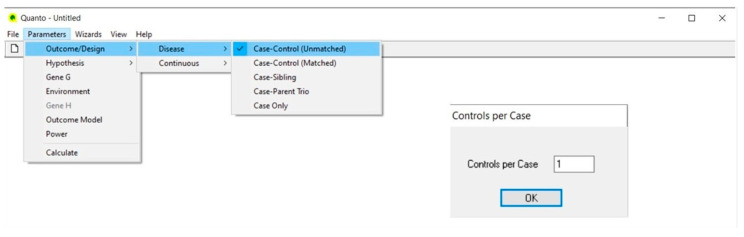
Outcome/design.

**Figure 2:**
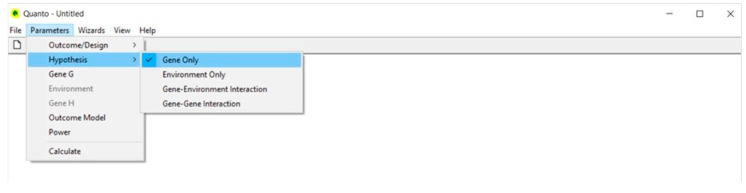
Hypothesis.

**Figure 3:**
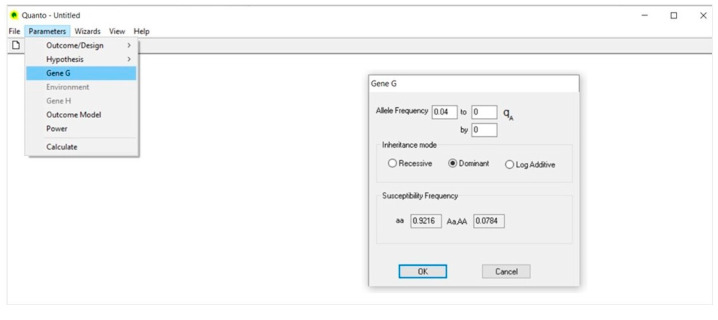
Gene G.

**Figure 4:**
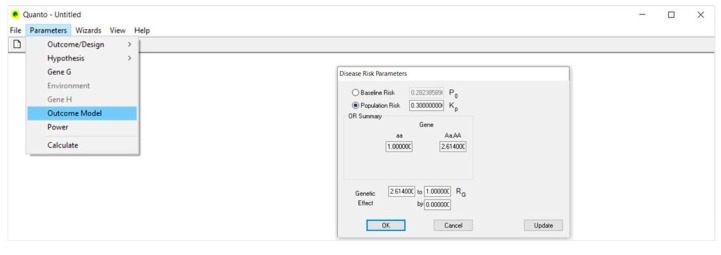
Outcome Model.

**Figure 5:**
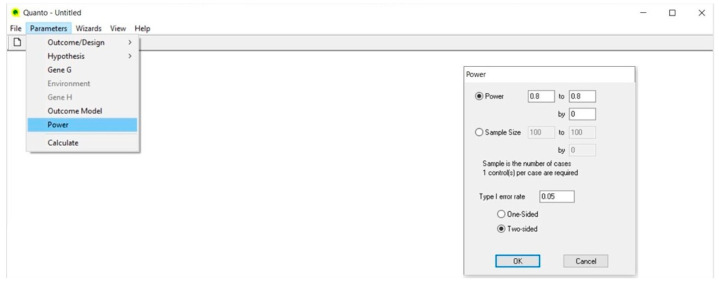
Power.

**Figure 6:**
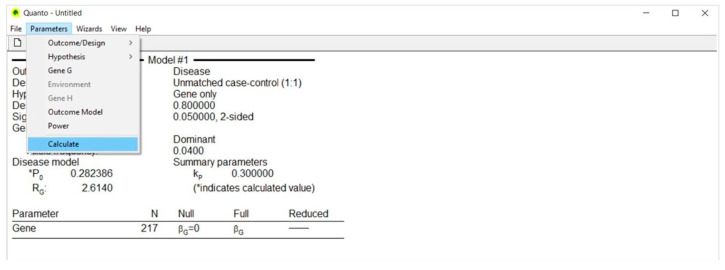
Calculate.

**Table 1:** Basic and genetic parameters for the sample size calculation in the genetic association studies.

		Definition
Basic	Alpha (Type I error or significance level)	The probability of incorrectly rejecting H_0_ according to sample results when it is actually true in the source population (*false positive*). The conventional value of the significance level is set at 0.05 indicating that there is a 5% probability of incorrectly rejecting the true H_0_.
	*Beta (Type II error)*	The probability of incorrectly retaining H_0_ according to sample results when it is false in the source population (*false negative*). The conventional value of this second kind of error is set at 0.20, meaning that the probability of failing to detect a statistically significant difference when it does exist, is no more than 20%.
	*Power (1-beta)*	The probability of correctly detecting a significant difference in the sample when it actually exists in the source population. Being the complement of beta, the conventional value of the power is 0.80, indicating that there is 80% probability of avoiding a false negative result.
**Genetic**	*Prevalence*	The proportion of individuals with the disease in a population at a given point in time. The smaller the proportion of affected subjects, the larger the sample size required.
	*Effect size*	The numeric value that indicates the strength of the association between a gene variant and a disease or the magnitude of the difference in the allelic/genotypic frequency between groups (i.e., cases and controls). For binary outcomes, it is expressed as a relative risk/odds ratio, while for continuous outcomes, as a percentage of the phenotypic variance. The smaller the effect size, the larger the sample size required.
	*Minor allele frequency (MAF)*	The frequency of the second most frequent allele for a polymorphism. According to MAF, a genetic variant can be classified as common (MAF > 5%), uncommon (1–5% MAF) and rare (MAF < 1%). The higher the MAF, the smaller the number of patients to be studied.
	*Linkage disequilibrium (LD)*	The non-random association of two or more alleles at different loci that occur together on the same haplotype more often than expected. The stronger the linkage between the marker allele and the disease allele, the higher the power of the study and the lower the sample size required to detect the gene–disease association.
	*Genetic inheritance model*	The model of inheritance of a genetic variant. Three genetic models are usually adopted: dominant, recessive and additive model. Genetic model misspecification reduces the study power and thus requires a higher sample size.
	*Errors*	They result from genotype/phenotype misclassification and/or missing genotype data. Their effect on the power loss cannot be quantified at the design stage of the study.

## Data Availability

Not applicable.
